# Scaling Language Models Show Diminishing Returns in Divergent Creativity

**DOI:** 10.3390/jintelligence14100244

**Published:** 2026-10-03

**Authors:** Yuemin Zhuo, Song Tong, Hongfei Du, Kaiping Peng

**Affiliations:** 1 Bay Area School of Applied Psychological Sciences, Beijing Normal University at Zhuhai, Zhuhai 519087, China; 202431061025@mail.bnu.edu.cn; 2 Beijing Key Laboratory of Applied Experimental Psychology, National Demonstration Center for Experimental Psychology Education (Beijing Normal University), Faculty of Psychology, Beijing Normal University, Beijing 100875, China; 3 Department of Psychological and Cognitive Sciences, Tsinghua University, Beijing 100084, China; pengkp@tsinghua.edu.cn

**Keywords:** scaling law, language models, divergent creativity

## Abstract

Recent advances in language models have revealed emerging capabilities in generating novel associations and exploring conceptual spaces, which are central processes underlying divergent creativity. However, as language models continue to scale up, a critical question remains unanswered: whether scaling can progressively enhance their capacity for divergent creativity. The relationship between model size and divergent creativity is our focus herein. In Study 1, we employed the Divergent Association Task to measure divergent word association. The results showed that larger language models demonstrated higher divergent creative performance. Notably, scaling language models yielded diminishing returns in the task. Study 2 assessed divergent creativity beyond word association, through a movie plot summary task, and also found that larger language models exhibited diminishing returns in the writing task. We further adjusted model temperatures as a robustness check, with the same pattern consistently replicated across both studies. These findings suggest that scaling alone may be insufficient to continuously improve the divergent creativity of language models, highlighting the importance of understanding their capabilities as they become increasingly integrated into human activities.

## 1. Introduction

Creativity has long been recognized as a hallmark of human cognition (Amabile, 1983; Brophy, 1998), which is central to human life and serves as a fundamental driver of technological innovation (Sawyer & Henriksen, 2024). It has catalyzed the progression of the Industrial Revolutions from the steam engine to the internal combustion engine, to electricity-driven production, and finally to computerized automation (Xu et al., 2018), all of which have significantly boosted productivity and efficiency. Each of these milestones was underpinned by creative breakthroughs that transformed daily life and paved the way for contemporary society.

Although the rapid advancement of generative artificial intelligence (AI) shows that artificial systems can also exhibit creative capabilities (Bellemare-Pepin et al., 2026; Gilhooly, 2024; Hubert et al., 2024; Koivisto & Grassini, 2023; Wang et al., 2025), little is known about how model size relates to divergent creativity in large language models (LLMs). Scaling laws suggest that model capabilities and performance improve as models increase in scale, such as the number of model parameters (i.e., model size; Li et al., 2025). However, scholars investigating creativity in LLMs using established creativity assessments have shown that larger models do not always outperform smaller ones. For example, Mistral-small with 24 billion parameters can sometimes outperform Mistral-large with 123 billion parameters in generating scientific ideas (Ruan et al., 2026). Nevertheless, most of the existing research has primarily focused on a limited number of frontier models, leaving unclear how creative performance changes as language models scale up.

In the current research, we examined 35 language models spanning 10 model families, including both open-source and closed-source models. By leveraging a broad spectrum of language models across different model parameters, we systematically investigated the relationship between model scaling and divergent creative performance. As LLMs are increasingly integrated into human–AI collaboration (Bai et al., 2025), examining the relationship between model size and divergent creativity not only advances our understanding of creativity in LLMs themselves but also provides insights into how LLMs may influence human creative cognition and creative work.

## 2. Related Work

Drawing on Guilford’s theory of creativity (Guilford, 1967), divergent creativity refers to the process by which individuals explore and think in multiple directions, which is widely regarded as an important component of creative cognition (H. Lee et al., 2023). Divergent creativity is considered central to cognition because it enables individuals to generate novel ideas and address open-ended problems (Brophy, 1998; Olson et al., 2021; Runco & Jaeger, 2012). It is widely accepted as a key indicator of creativity as it captures the initial, generative phase of the creative process, during which many ideas are generated before their usefulness is evaluated (Bellemare-Pepin et al., 2026). Consistent with this, divergent creativity performance has been shown to predict real-world creative achievement (Said-Metwaly et al., 2024).

Divergent creativity is commonly measured using the Alternative Uses Task (AUT), in which participants are asked to generate novel uses for everyday objects and their responses are evaluated in terms of novelty (Vartanian et al., 2019). For example, in the AUT, participants may be asked to generate as many novel uses as possible for a common object, such as a brick. A typical response might be “use bricks to build a house”, whereas a more creative response might be “use a brick as a paperweight”. Scoring the AUT typically requires raters to manually evaluate responses, which largely depends on raters’ subjective perception of creativity. Moreover, the task is constrained by a handful of everyday objects, which may confound creativity with familiarity or pre-existing associations with particular objects (H. Lee et al., 2023).

To automatically assess and quantify divergent creativity, Olson et al. (2021) introduced the Divergent Association Task (DAT), which asks participants to generate ten words that are as different as possible from one another across all their meanings and uses. More creative responses consist of word pairs with greater semantic distance, for example, rosemary and prickle rather than shoes and soccer. One might argue, however, that word-based tasks alone can hardly capture the full picture of creativity in language models. A complementary approach evaluates creativity through creative writing which is based on sentence-level text. Past work has used the generation of movie synopses based on a given title to assess creative writing (Bellemare-Pepin et al., 2026; Marco et al., 2025).

Divergent creativity in these tasks is primarily computed by semantic distance, which reflects the relationships between word embeddings (Charlesworth et al., 2023). Specifically, semantic distance is typically calculated from the cosine similarity between words (K. Zhou et al., 2022), the cosine of the angle between them, such that a greater distance reflects a more remote relationship and thus greater creativity. In the DAT, divergent creativity is indicated by the semantic distance between generated word pairs. As mentioned above, the semantic distance between rosemary and prickle is greater than that between shoes and soccer, suggesting that the former word pair represents greater creativity than the latter. Additionally, for the writing task, Divergent Semantic Integration (DSI; Johnson et al., 2022) quantifies divergent creativity as the semantic distance among words in a narrative, allowing the computation of contextual word embeddings. Taken together, these methodological advances make it possible to measure and evaluate divergent creativity automatically and quantitatively, without relying on human judges whose ratings may introduce bias.

Since the emergence of language models as one of the cutting-edge technologies in generative AI, particularly given their notable capabilities in problem solving and reasoning (Guo et al., 2025), scholars have examined whether language models can also display creativity. A growing body of research has studied the creativity of language models and compared it with that of humans (Bellemare-Pepin et al., 2026; Gilhooly, 2024; Hubert et al., 2024; Koivisto & Grassini, 2023; Wang et al., 2025), demonstrating that language models can exhibit creativity and can match average human performance on creativity tasks, including both remote word association and writing tasks. Although language models have been shown to exhibit creativity, our understanding of how model size (i.e., the number of parameters) relates to creativity remains nascent.

Scaling laws have been proposed to characterize how the performance of language models scales with model size (number of parameters), dataset size (number of pretraining tokens), and computational budget (Li et al., 2025). In the present work, we focus primarily on model size. Technologically, language models tend to perform better as the number of parameters increases, because their test loss, a metric of how well a model performs, decreases (Li et al., 2025). GPT-3 can solve few-shot tasks through in-context learning, whereas smaller models may not (Binz & Schulz, 2023). Nevertheless, the relationship between model parameters and performance is not always linear. Scaling patterns may vary depending on the tasks. Prior research has documented U-shaped scaling on some tasks (e.g., Sig Figs, which requires language models to round numbers based on the specified number of decimal places), where model performance initially declines as model size increases but improves after reaching a certain point (Wei et al., 2023).

In terms of creativity, few studies have systematically explored the relationship between model parameters and divergent creativity. Existing literature provides preliminary evidence that small language models can also outperform larger models on some specific creative tasks (Marco et al., 2025; Ruan et al., 2026). In a writing task in which models generate movie synopses based on given titles, language models such as ChatGPT-3.5 and ChatGPT-4o are better at telling a logical story with fewer mistakes, whereas a fine-tuned smaller language model (i.e., BART-large) can surprisingly produce more novel content than ChatGPT (Marco et al., 2025). Similarly, Ruan et al. (2026) found that Mistral-small, despite having substantially fewer parameters (24 billion), slightly outperformed Mistral-large (123 billion parameters) in generating scientific ideas.

While these empirical results provide initial evidence that small language models can sometimes outperform the larger ones, these studies tested only a limited number of models, thereby limiting our understanding of whether this pattern holds in a broader spectrum of language models from different model families. More importantly, guided by scaling laws, AI companies keep scaling up their language models, which leads to an open question: whether larger language models persistently yield greater divergent creativity, or whether increasingly bigger models show diminishing returns in divergent creativity. To answer these questions, using the DAT and a movie plot summary task, we evaluated 35 language models to comprehensively investigate the relationship between language model parameters and divergent creativity.

To probe the association between language model parameters and divergent creativity, we used two tasks that assess divergent creativity across two studies, including word generation and writing. In Study 1, we employed the DAT to examine the relationship between model size and divergent creativity, testing (a) whether language models exhibit greater divergent creativity as model size increases and (b) whether this divergent creativity shows diminishing returns at larger model sizes. Building on Study 1, Study 2 employed a movie plot summary task to measure divergent creativity beyond word generation alone, seeking to replicate the diminishing returns results in creative writing. Because prior studies have shown that model temperature affects the degree of randomness in output, we also adjusted model temperature as a robustness check in both studies. The temperature parameter typically ranges from 0 to 1: higher values, such as 0.8, make the output more random, whereas lower values, such as 0.2, make it more focused and deterministic (OpenAI, 2026).

## 3. Study 1

Using the DAT, we began by examining whether larger language models show greater divergent creativity. More importantly, we tested whether scaling up language models exhibits diminishing returns.

### 3.1. Method

#### 3.1.1. Language Models

In Study 1, we selected 35 language models spanning a wide range of model sizes from 14 million to 2.4 trillion parameters, drawn from the Pythia, Qwen, ERNIE, DeepSeek, ChatGPT, Claude, Llama, GLM, Kimi, and Mistral families (see Table 1 for a detailed summary). All models were called through their application programming interface (API), except for Pythia which we downloaded from Hugging Face^1^.

#### 3.1.2. Divergent Association Task

To enhance the replicability and objectivity of divergent creativity assessment in language models, we used the DAT which is a validated measure that captures the ability to generate and combine remote concepts in semantic space. In Study 1, both language models and human participants completed the DAT, listing ten words that are as different from one another as possible across all their meanings and uses (Olson et al., 2021, 2023; see Supplementary Materials for the prompt). To generate a number of model responses approximately equivalent to the human sample size, we requested each model to respond to this task independently 700 times using the API.

#### 3.1.3. Human Benchmark

In Study 1, our pooled dataset included 26,585 human participants from 159 countries or regions (*M*_age_ = 41.38, *SD*_age_ = 16.40; 15,889 female). Each participant completed the DAT and the mean divergent creativity score was 80.86 (*SD* = 6.58), which served as an average human benchmark to provide a reference point for visualizing language model performance.

#### 3.1.4. Analytical Strategy

Divergent Creativity Score Calculation. Before calculating creativity scores, we preprocessed the generated responses by removing invalid outputs, including non-word responses. To calculate the cosine similarity between all possible pairs among the ten words generated in the DAT, we used GloVe (Pennington et al., 2014), which is based on global word–word co-occurrence statistics and was pretrained on the Common Crawl corpus of 840 billion tokens. GloVe provides an effective method for measuring the cosine similarity between words. All responses generated by different language models were evaluated using GloVe. To be specific, for each response generated by a language model, we calculated the cosine similarity between every possible pair of word embeddings and averaged these values to obtain the overall semantic similarity score. The average semantic similarity score was multiplied by 100 and subtracted from 100 to create the divergent creativity score. The equations are presented as follows:

Equation (1):

(1) $$Sim\;(v_{i},\;v_{j})=\frac{v_{i}\cdot v_{j}}{\left||v_{i}||\;||v_{j}|\right|}$$

where *v_i_* and *v_j_* denote the GloVe vectors of the *i*-th and *j*-th words in a response, v*_i_* ⋅ v*_j_* represents the dot product, and ||*v_i_*|| ||*v_j_*|| represents the product of their Euclidean norms.

Equation (2):

(2) $$Divergent\;Creativity\;Score=100-100\left(\frac{2}{N(N-1)}\sum_{i<j}Sim\;(v_{i},\;v_{j})\right)$$

where the summation runs over all *N*(*N* − 1)/2 unique word pairs (i.e., 45 pairs when *N* = 10).

Statistical Method. Due to the fact that model parameters ranged from millions to trillions of parameters across different model families, we log-transformed the model parameters and controlled for model family fixed effects (i.e., *K* − 1 model family dummy variables). To quantify the pattern of the relationship between model parameters and creativity, we performed the following analysis:

Equation (3):

(3) $${Divergent\;Creativity\;Score}_{i}=\beta_{0}+{\beta_{1}{Log}_{10}(Model\;Parameters)}_{i}+\;\beta_{2}{Log}_{10}{{\left(Model\;Parameters\right)}^{2}}_{i}+\sum_{k=1}^{K-1}\beta_{3}{Model\;Families}_{ik}+\epsilon_{i}$$

where *i* represents each response generated by a language model, *k* denotes each language model, *K* − 1 represents model family dummy variables that account for all model families except the reference group, and *ϵ* represents the residual error.

### 3.2. Results and Discussion

**Figure 1:**
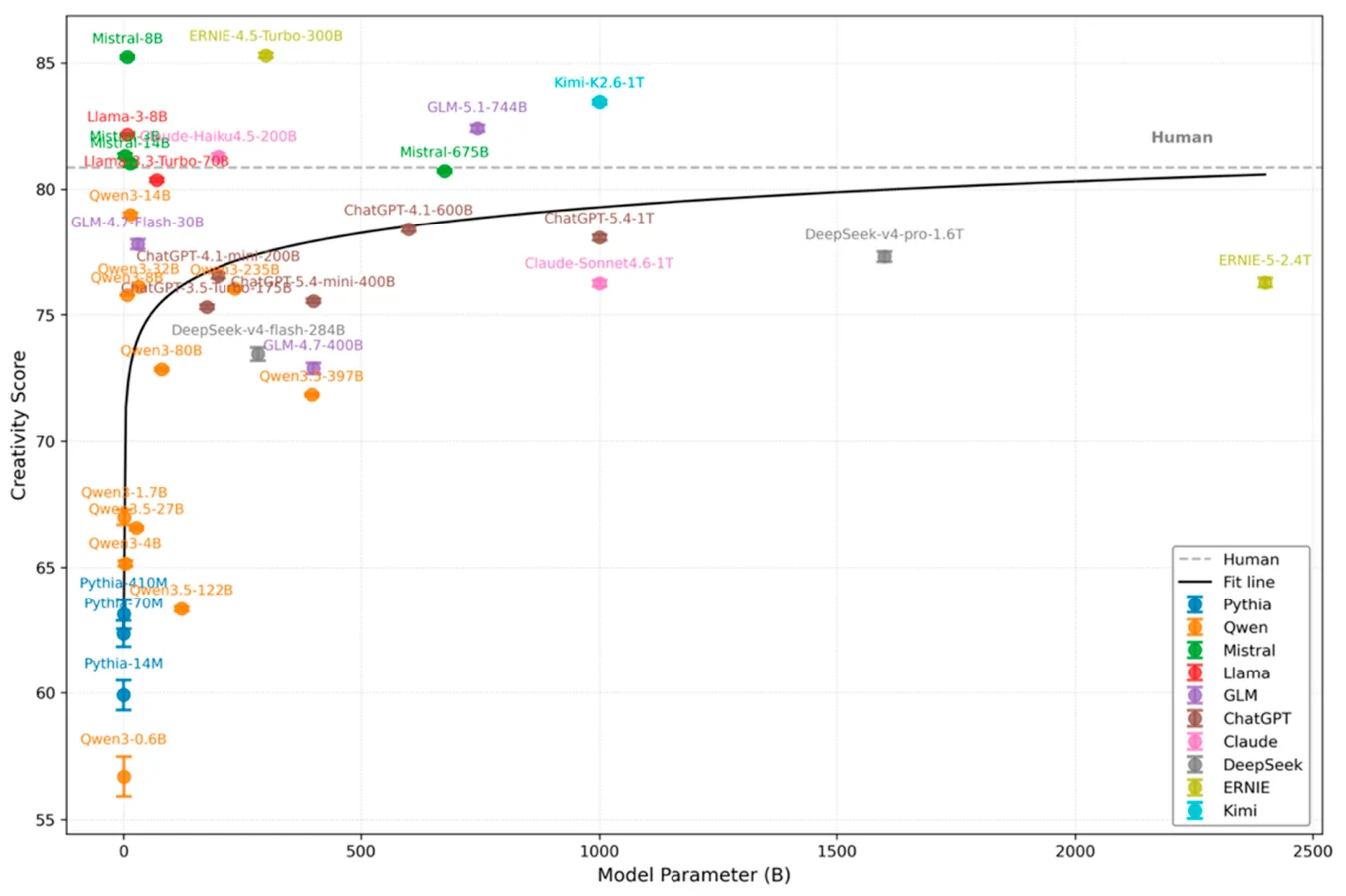
Study 1: DAT results.

In Study 1, the multiple regression analysis indicated that language models showed higher levels of divergent creativity as model size increased, *B* = 1.52, *SE* = 0.08, *p* < .001, 95% CI [1.35, 1.68], AIC^2^ = 169,052.7, BIC = 169,150.0, while controlling for model family fixed effects (see Figure 1 and Table 2 for the detailed results). Notably, we further incorporated a quadratic term for the log-transformed model parameters. The results suggested a better fit, indicating that the divergent creativity performance diminished as model size increased, *B* = −2.87, *SE* = 0.08, *p* < .001, 95% CI [−3.02, −2.72], AIC = 167,717.0, BIC = 167,822.3 (see also Figure 1 and Table 2).

In the statistical analysis, we further conducted a series of robustness checks. Foremost, we adjusted model temperatures^3^, and the results remained unchanged (see also Table 3). To examine whether closed-source models influenced the observed diminishing returns effect, we conducted sensitivity analyses excluding language models from OpenAI and Anthropic. The results remained robust (see details in Appendix A). Additionally, because each language model generated multiple responses while model size remained constant within each model, we conducted model-level analyses. The results replicated our findings (see details in Appendix B).

Study 1 provided initial evidence that scaling up language models is associated with greater divergent creativity. Extending this, the results also revealed that language models exhibited diminishing returns in divergent creativity as model parameters increased; this pattern held across a series of robustness checks involving different temperature settings and a supplementary model-level analysis. These findings suggest that the divergent creativity of language models may not be contingent on model size alone.

## 4. Study 2

Extending Study 1, we examined whether larger language models show diminishing returns in divergent creativity on a more complex task. Specifically, we used a writing task that required models to generate movie synopses from given titles, because word generation alone may not capture the text-generation abilities of language models.

### 4.1. Method

#### 4.1.1. Language Models

The language models used in Study 2 were identical to those in Study 1. Apart from the Pythia models which were downloaded in the local environment, we accessed all models through the API.

#### 4.1.2. Movie Plot Summary Task

In Study 2, the movie dataset contained 250 real-world existing movies, including their titles and release years (Laurito et al., 2025; see Supplementary Materials for the prompt). Each model was asked to generate a plot summary for each movie based on its title and release year, ensuring comparable dataset sizes between the creative writing samples produced by language models and those produced by humans.

#### 4.1.3. Human Benchmark

The human benchmark in Study 2 consisted of 250 movie plot summaries, each written by a different human participant. The average divergent creativity score for these summaries was 72.60 (*SD*_movie_ = 0.87), serving as the human benchmark to provide a reference point for visualizing language model performance.

#### 4.1.4. Analytical Strategy

Divergent Creativity Score Calculation. Prior to analysis, text preprocessing was conducted to standardize the textual data. Specifically, all text was converted to lowercase, tokenized into words, and stripped of common English stop words using the stop word list provided by the natural language toolkit (nltk) package in Python 3.9.13. These steps reduced linguistic noise and retained semantically meaningful words for subsequent analysis. To compute Divergent Semantic Integration (DSI) scores for the creative writing (Johnson et al., 2022), we used OpenAI’s text-embedding-3-large^4^ to obtain word embeddings for each movie summary. All responses generated by different language models were evaluated using the text-embedding-3-large model. Specifically, we calculated the cosine similarity between all possible words in each summary, averaged these values, multiplied the average by 100, and subtracted the result from 100. Scores ranged from 0 to 100, with higher scores indicating greater divergent creativity.

Statistical Method. The statistical method in Study 2 was the same as in Study 1.

### 4.2. Results and Discussion

**Figure 2:**
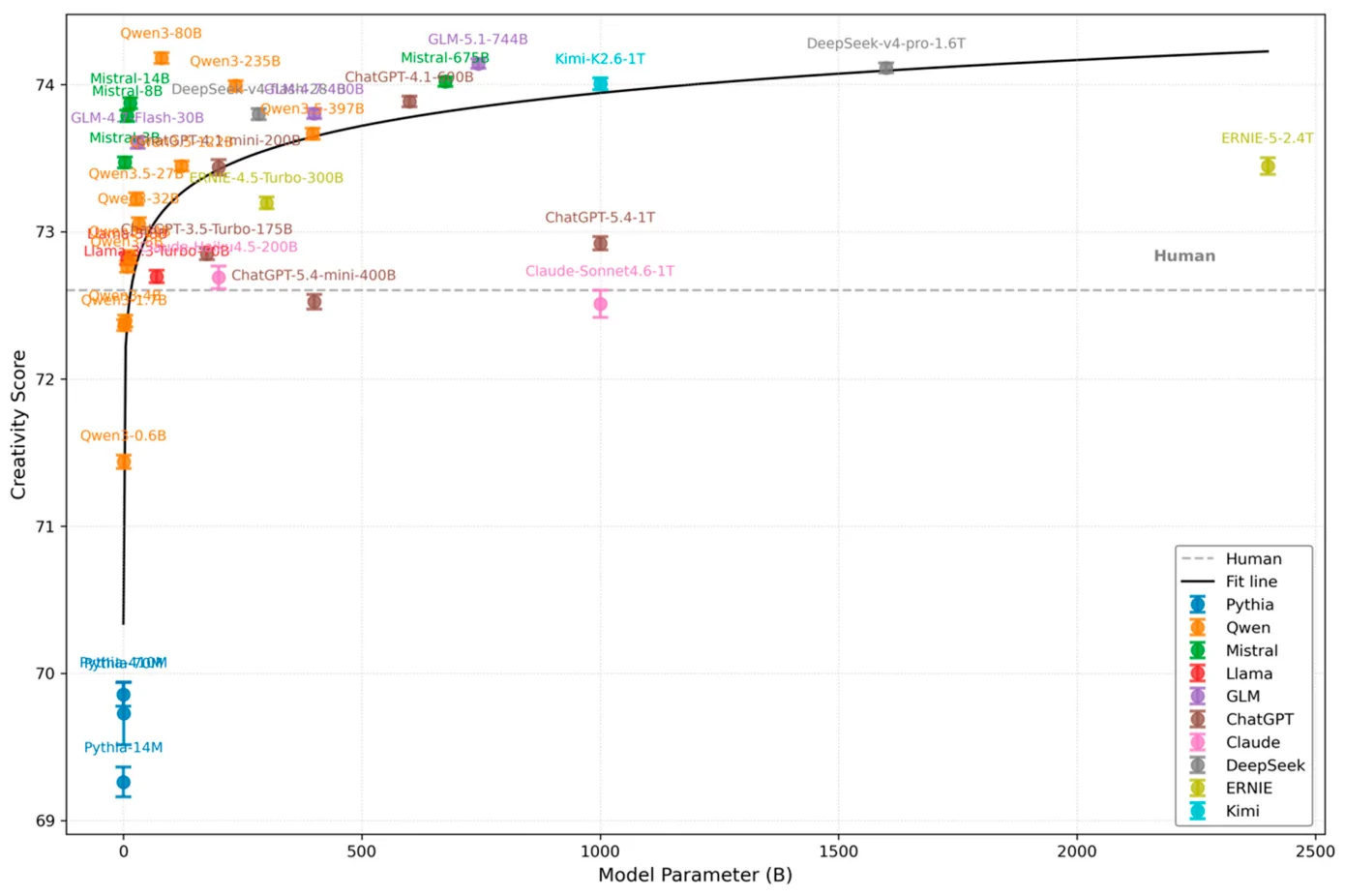
Study 2: Movie plot summary results.

In Study 2, a multiple regression analysis controlling for model family fixed effects indicated that larger language models also displayed diminishing returns in creativity, *B* = −0.29, *SE* = 0.02, *p* < .001, 95% CI [−0.33, −0.26] (see Figure 2 and Table 4 for results). These results remained robust when we adjusted model temperatures (see Table 5). The diminishing returns effect was replicated after excluding closed-source models and conducting model-level analyses (see details in Appendix A and Appendix B).

Although Study 1 relied on word generation, which some might argue does not fully capture the essence of creativity, Study 2 addressed this limitation by using a writing task. Importantly, the results again revealed that larger language models yielded diminishing returns in divergent creativity. Furthermore, we changed the embedding tool from GloVe to OpenAI’s text-embedding-3-large to ensure the robustness of our findings.

## 5. General Discussion

Language models are now capable of generating novel associations. However, little research has systematically investigated the relationship between language model size and divergent creativity. Our results suggest that language models exhibit greater divergent creativity as the number of parameters increases. Remarkably, we also found that larger language models show diminishing returns in divergent creativity across both word association and writing tasks. These results remained robust after controlling for model family fixed effects and adjusting model temperature. Our findings underscore that scaling language models may not progressively enhance divergent creativity, challenging the prevailing assumption that bigger models are inherently more capable.

### 5.1. Contributions and Implications

The current research shows that larger language models exhibit greater divergent creativity, carrying implications for both language models and creativity. With respect to language models, although the number of pretraining tokens does not strictly increase with the number of parameters, the two are highly correlated because a model with far more parameters than pretraining tokens tends to overfit (Li et al., 2025). More parameters therefore usually indicate that a model has been pretrained on a larger amount of data (Li et al., 2025). Scaling up model parameters may thus enhance a model’s corpus-based knowledge and representational capacity, allowing it to draw on a more extensive reservoir of linguistic patterns and knowledge.

Regarding creativity, associative theory posits that creativity is an associative process involving the formation of new combinations (Beaty & Kenett, 2023). A larger body of knowledge naturally affords a greater number of possible combinations within the knowledge space. By leveraging language models ranging from small to large in scale, our research empirically corroborates the theory that a larger repertoire of associations increases the probability of generating creative combinations. Furthermore, consistent with previous findings (Wang et al., 2025), our research revealed that divergent creative performance can emerge from artificial systems.

Critically, beyond showing that larger models exhibit greater creativity, our research demonstrates that they also show diminishing returns as model parameters increase. These findings resonate with previous research showing that smaller language models can outperform larger ones on certain tasks (Marco et al., 2025; Ruan et al., 2026). One possible explanation is that language models are trained largely on vast amounts of human-generated text, yet the stock of public text and data on the internet may be nearly exhausted (Epoch AI, 2023). A second explanation concerns technical details, namely that larger models increasingly adopt Mixture-of-Experts architectures (Masoudnia & Ebrahimpour, 2014), which activate only a small batch of parameters for a given task and may therefore constrain performance. Moreover, larger language models typically undergo extensive post-training alignment procedures, such as reinforcement learning from human feedback (RLHF; Sivan et al., 2024), which may influence their outputs.

Our findings have implications at both technological and societal levels. At the technological level, scaling language model size produces progressively smaller gains in divergent creativity, suggesting that the improvement may ultimately be bounded by limitations in model parameters. In other words, this constraint is inherent to language model technology itself (Zhao et al., 2025), independent of who uses it. At the societal level, the ability to access more advanced models is not equally distributed, reflecting the digital divide (Capraro et al., 2024; Khowaja et al., 2024). Because the frontier language models require substantial computational infrastructure and financial investment, they may remain accessible primarily to individuals or organizations with greater resources, such as wealthier users and large corporations. The emerging divide highlights a broader implication of the diminishing returns results. As further gains become increasingly costly, expanding access to capable and efficient mid-sized models may offer a more equitable approach to distribute the benefits of AI, rather than restricting these advantages to those with greater resources.

### 5.2. Limitations and Future Perspectives

First, we predominantly assessed divergent creativity in the present study. Creativity, however, is multifaceted and also encompasses convergent creativity, which emphasizes the ability to identify the optimal solution to a well-defined problem (Guilford, 1967). Convergent creativity is often measured with tasks such as the Bridge-the-Associative-Gap task, for instance, if presented with cheese and cat, participants may provide “mouse” as the most appropriate bridge word (Gianotti et al., 2001). We did not assess convergent creativity in the present research, partially because such tasks typically rely on a fixed set of items and are less scalable than the DAT. Furthermore, according to the creativity diamond framework (Childs et al., 2022), creativity can be further disentangled into more nuanced categories, including morphological analysis, analogical reasoning, critical thinking, and more. Future research would benefit from investigating other forms of creativity which could enrich and extend our findings.

Another limitation is that our study relied on English language input and output. We encourage future research to test whether these findings remain robust across other languages. Relatedly, language models, particularly those developed in the United States, are trained primarily on English-dominant and Western-centric corpora, and our research did not examine differences across languages and cultures. This echoes a longstanding critique in psychological science in terms of the overrepresentation of Western, Educated, Industrialized, Rich and Democratic (WEIRD) populations in research, which limits the generalizability of research findings to broader human populations (Henrich et al., 2010; Medvedev et al., 2024). Consistent with this concern, the training corpora underlying many language models disproportionately overrepresent English and Western textual content. Therefore, the diminishing returns observed in divergent creativity here may reflect the effect of scaling language model size as evaluated within a culturally narrow frame, rather than a universally applicable property (Lu et al., 2025). Beyond relying on embedding metrics that are derived from WEIRD cultures, future research should consider developing embedding metrics that incorporate more culturally diverse representations. Future research could also investigate whether the diminishing returns pattern generalizes across languages and cultures.

One additional limitation pertains to the tasks, in which language models were asked to generate words and to write movie plots based on film titles and release years. Although DAT scores were computed using GloVe embeddings trained on internet data in Study 1, semantically inappropriate outputs could potentially influence the estimated divergent creativity scores because we did not independently verify that every generated response constituted a meaningful word. Unlike word generation where model responses were drawn from an open combinatorial space, movie plot responses may not entirely reflect semantic space exploration. Instead, model outputs may partly draw on movie information encountered during pretraining, if such information was included in the model’s training data. Future research could address this by using fictitious or newly generated movie titles, which would reduce the potential influence of prior exposure to existing data. In addition to word association and writing tasks, which are linguistically based, creativity can be expressed more broadly through the arts (E. Zhou & Lee, 2024), such as painting, sculpture, and design, and through the sciences, such as technological inventions. For example, as a landmark event at the intersection of AI and art, the Théâtre D’opéra Spatial, created using Midjourney, won the 2022 Colorado State Fair’s annual fine art competition. Future research could leverage multimodal models to analyze images and videos, thereby extending the study of creativity beyond text-based tasks.

Prompts are also central to model output and divergent creativity (Hu & Collier, 2024). Our robustness checks mainly focused on a model setting, namely, temperature, that affect model outputs. However, the prompt may also influence the divergent creativity. Empirical evidence suggests that when different persona-based prompts, such as gender, age, and historically renowned creative figures, are specified, model outputs can change substantially (Pataranutaporn et al., 2025; Wang et al., 2025). Because our study held the prompt constant across models, we cannot determine whether persona-based prompts would influence our diminishing returns results, or whether such prompts might interact with model parameters to produce different creativity outcomes. Future research could systematically test whether our patterns still hold by varying prompts.

Although our research systematically examined language models across different families, it did not cover the full spectrum of available models. First, some frontier models are not publicly available, which makes their creativity difficult to evaluate. For example, Anthropic Mythos may contain up to 10 trillion parameters but is not publicly available (Claude, 2026). Second, although our results remained robust when we excluded closed-source models and re-ran the analyses, the parameter counts of closed-source models were based on approximate estimates rather than publicly disclosed. Therefore, the observed relationship between model parameters and divergent creativity should be interpreted with caution, particularly regarding the precise magnitude of scaling effects. Future research should aim to include a broader range of models, particularly those at the frontier of scale with publicly available specifications.

In light of future directions, a meaningful avenue for future research is to examine how scaling language models affects human creativity. Research attention has increasingly shifted toward human–AI collaboration which reveals that AI can play a role in shaping human creativity (Cheng & Zhang, 2025; Doshi & Hauser, 2024; Rafner et al., 2023). For example, AI can act as a catalyst that enhances the novelty, usefulness, and quality of ideas, particularly for less creative individuals, thereby expanding human creative capacity (B. C. Lee & Chung, 2024). Building on these findings, future research could investigate whether larger models exert a progressively greater influence on human creativity, or whether the effect of scaling on human creative performance likewise exhibits diminishing returns (Hackenburg et al., 2025).

According to the present findings, if diminishing returns extend beyond divergent creativity to other domains, the marginal benefits of accessing increasingly larger frontier models may become limited. In educational and workplace settings, our findings are consistent with previous research suggesting that smaller language models are not always outperformed by larger models in certain tasks (Ruan et al., 2026), and offer new insights into personalized human–AI collaboration (Kirk et al., 2024), such as mid-sized models fine-tuned to users’ specific goals or cognitive styles. This does not imply that frontier models should be devalued or replaced; rather, personalized models may provide more adaptive support for diverse users as a complementary approach to frontier models. 

## Figures and Tables

**Table 1:** Language model details.

Model Family	Model Parameters	Availability	Temperature Range
Pythia	14M, 70M, 410M	Open-weight	do_sample = True or False
Qwen	0.6B(3), 1.7B(3), 4B(3), 8B(3), 14B(3), 27B(3.5), 32B(3), 80B(3), 122B(3.5), 235B(3), 397B(3.5)	Open-weight	0–2
ERNIE	300B(4.5-turbo), 2.4T(5)	Open-weight	0–2
DeepSeek	284B(V4-flash), 1.6T(V4-pro)	Open-weight	0–2
Llama	8B(3), 70B(3.3)	Open-weight	0–1
GLM	30B(4.7-flash), 400B(4.7), 744B(5.1)	Open-weight	0–2
Kimi	1T(2.6)	Open-weight	0.6
Mistral	3B(3), 8B(3), 14B(3), 675B(3)	Open-weight	0–1
ChatGPT	1T(5.4), 400B(5.4-mini), 600B(4.1), 200B(4.1-mini), 175B(3.5-turbo)	Closed-source	0–1
Claude	1T(Sonnet 4.6), 200B(Haiku 4.5)	Closed-source	0–1

Note. M, B, and T denote million, billion, and trillion, respectively. For closed-source models, we assumed parameter values for visualization and analysis based on existing estimates: 1 trillion parameters for ChatGPT 5.4 and Claude Sonnet 4.6; 600 billion for ChatGPT 4.1; 400 billion for ChatGPT 5.4-mini; and 200 billion for ChatGPT 4.1-mini and Claude Haiku 4.5. Estimated parameter values were included to provide an approximate indicator of model scale; however, these values should not be interpreted as exact measurements of model parameters. In Pythia, do_sample = True means that the model generates responses by sampling from the probability distribution, making outputs more diverse and less deterministic. do_sample = False means that the model uses deterministic sampling, typically selecting the most likely next token, which makes the output more stable and reproducible. For Kimi, the temperature was fixed at 0.6 and could not be adjusted.

**Table 2:** Study 1: DAT scores as the outcome variable.

LLMs Temperature: Default	Model 1: Linear				Model 2: Quadratic			
	*B*	*SE*	*p*	95% CI	*B*	*SE*	*p*	95% CI
Intercept	72.85	0.25	<.001	[72.36, 73.34]	66.80	0.29	<.001	[66.23, 67.38]
Log_10_Parameter	1.52	0.08	<.001	[1.35, 1.68]	11.37	0.28	<.001	[10.82, 11.91]
Log_10_Parameter^2^	NA	NA	NA	NA	−2.87	0.08	<.001	[−3.02, −2.72]
Claude	1.90	0.24	<.001	[1.42, 2.37]	2.35	0.23	<.001	[1.89, 2.81]
DeepSeek	−1.77	0.24	<.001	[−2.24, −1.29]	−0.21	0.24	.379	[−0.68, 0.26]
ERNIE	3.50	0.24	<.001	[3.02, 3.97]	5.90	0.24	<.001	[5.42, 6.38]
GLM	1.33	0.21	<.001	[0.91, 1.74]	1.00	0.21	<.001	[0.60, 1.41]
Kimi	6.05	0.32	<.001	[5.43, 6.68]	8.38	0.32	<.001	[7.76, 9.00]
Llama	6.28	0.26	<.001	[5.77, 6.79]	4.74	0.26	<.001	[4.23, 5.24]
Mistral	7.12	0.22	<.001	[6.69, 7.54]	7.12	0.21	<.001	[6.70, 7.53]
Pythia	−11.14	0.30	<.001	[−11.73, −10.56]	−5.68	0.32	<.001	[−6.32, −5.05]
Qwen	−4.94	0.18	<.001	[−5.31, −4.58]	−5.43	0.18	<.001	[−5.79, −5.08]
AIC	169,052.7				167,717.0			
BIC	169,150.0				167,822.3			

Note. NA indicates that the term was not included in the model, and CI denotes confidence interval. Model predictors represent dummy variables for model families.

**Table 3:** Study 1: Comparison of different model fit metrics and adjusted model temperature.

Models	*B*	*SE*	*p*	95% CI	AIC	BIC
Temperature: default						
Study 1: DAT						
Model 1: Linear	1.52	0.08	<.001	[1.35, 1.68]	169,052.7	169,150.0
Model 2: Quadratic	−2.87	0.08	<.001	[−3.02, −2.72]	167,717.0	167,822.3
Temperature: low						
Study 1: DAT						
Model 1: Linear	2.58	0.06	<.001	[2.47, 2.70]	152,477.6	152,574.9
Model 2: Quadratic	−3.47	0.05	<.001	[−3.57, −3.37]	148,419.8	148,525.2
Temperature: high						
Study 1: DAT						
Model 1: Linear	3.04	0.10	<.001	[2.84, 3.24]	179,292.1	179,389.4
Model 2: Quadratic	−2.72	0.10	<.001	[−2.91, −2.53]	178,511.7	178,617.1

**Table 4:** Study 2: Movie plot summary task scores as the outcome variable.

LLMs Temperature: Default	Model 1: Quadratic			
	*B*	*SE*	*p*	95% CI
Intercept	71.03	0.07	<.001	[70.90, 71.16]
Log_10_Parameter	1.58	0.06	<.001	[1.46, 1.70]
Log_10_Parameter^2^	−0.29	0.02	<.001	[−0.33, −0.26]
Claude	−0.51	0.05	<.001	[−0.62, −0.41]
DeepSeek	0.85	0.05	<.001	[0.75, 0.96]
ERNIE	0.25	0.05	<.001	[0.14, 0.36]
GLM	0.84	0.05	<.001	[0.75, 0.93]
Kimi	0.88	0.07	<.001	[0.74, 1.02]
Llama	0.15	0.06	.009	[0.04, 0.26]
Mistral	1.34	0.05	<.001	[1.25, 1.44]
Pythia	−1.51	0.07	<.001	[−1.65, −1.36]
Qwen	0.53	0.04	<.001	[0.46, 0.61]
AIC	24,571.7			
BIC	24,663.6			

Note. CI denotes confidence interval. Model predictors represent dummy variables for model families.

**Table 5:** Study 2: Comparison of different model fit metrics and adjusted model temperature.

Models	*B*	*SE*	*p*	95% CI	AIC	BIC
Temperature: default						
Study 2: Movie						
Model 1: Linear	0.57	0.02	<.001	[0.53, 0.61]	24,857.1	24,942.0
Model 2: Quadratic	−0.29	0.02	<.001	[−0.33, −0.26]	24,571.7	24,663.6
Temperature: low						
Study 2: Movie						
Model 1: Linear	0.56	0.02	<.001	[0.52, 0.60]	24,750.5	24,835.4
Model 2: Quadratic	−0.28	0.02	<.001	[−0.31, −0.24]	24,499.3	24,591.3
Temperature: high						
Study 2: Movie						
Model 1: Linear	0.42	0.02	<.001	[0.38, 0.46]	26,419.4	26,504.3
Model 2: Quadratic	−0.52	0.02	<.001	[−0.56, −0.49]	25,639.5	25,731.4

## Data Availability

All data are available at Open Science Framework: https://osf.io/wt32k/overview?view_only=b6d9fc9c106a4012b1f22bcb9620803b (accessed on 2 August 2026).

## Notes

1 Pythia 14M is available at: https://huggingface.co/EleutherAI/pythia-14m (accessed on 18 May 2026); Pythia 70M is available at: https://huggingface.co/EleutherAI/pythia-70m (accessed on 18 May 2026); Pythia 410M is available at: https://huggingface.co/EleutherAI/pythia-410m (accessed on 18 May 2026).

2 Akaike Information Criterion (AIC) and Bayesian Information Criterion (BIC) are indices that evaluate the relative fit of competing models while penalizing model complexity. Lower AIC and BIC values indicate a better balance between goodness of fit and model complexity (Vrieze, 2012).

3 As presented in Table 1, the available temperature ranges varied across model families because different model families used different temperature scales. For example, Qwen models allowed temperatures ranging from 0 to 2, whereas Mistral models supported temperatures ranging from 0 to 1. Therefore, in this and the following study, the “low” temperature refers to the lowest temperature value available for each model, while the “high” temperature refers to the highest temperature value available for each model. In doing so, we examined whether more deterministic versus more stochastic settings would influence our findings.

4 The text-embedding-3-large is an embedding model for both English and non-English tasks from OpenAI https://developers.openai.com/api/docs/models/text-embedding-3-large (accessed on 22 May 2026). Embeddings are a numerical representations of text that can be used to measure the relatedness between two pieces of text.

## Supplementary Materials

Supplementary Materials are available at Open Science Framework: https://osf.io/wt32k/overview?view_only=b6d9fc9c106a4012b1f22bcb9620803b (accessed on 28 September 2026).

## Abbreviations

The following abbreviations are used in this manuscript:

AI	Artificial Intelligence
LLMs	Large Language Models
DAT	Divergent Association Task
AUT	Alternative Uses Task
DSI	Divergent Semantic Integration
API	Application Programming Interface
RLHF	Reinforcement Learning from Human Feedback

## Appendix A. Sensitivity Analysis

Given the proprietary nature of the ChatGPT and Claude model families and the fact that their developers have not disclosed detailed technical reports, we estimated the model sizes of these models in the main analyses. Therefore, we conducted a sensitivity analysis by excluding OpenAI and Anthropic models from the statistical analyses to examine whether these closed-source models influenced the observed diminishing returns in divergent creativity.

In Study 1, as shown in Table A1, the quadratic model showed relatively lower AIC and BIC values than the linear model, suggesting that larger language models yielded diminishing returns in creativity even after excluding OpenAI and Anthropic models. The same pattern of results was observed in Study 2, in which the quadratic model also demonstrated relatively lower AIC and BIC values (see Table A2).

**Table A1:** Study 1: Sensitivity analysis.

Models	*B*	*SE*	*p*	95% CI	AIC	BIC
Temperature: default						
Study 1: DAT						
Model 1: Linear	1.64	0.09	<.001	[1.45, 1.82]	138,877.4	138,956.2
Model 2: Quadratic	−3.22	0.09	<.001	[−3.40, −3.04]	137,718.7	137,805.4
Temperature: low						
Study 1: DAT						
Model 1: Linear	2.77	0.07	<.001	[2.64, 2.90]	125,291.3	125,370.1
Model 2: Quadratic	−3.82	0.06	<.001	[−3.94, −3.69]	121,844.2	121,930.9
Temperature: high						
Study 1: DAT						
Model 1: Linear	3.28	0.12	<.001	[3.05, 3.51]	147,245.7	147,324.5
Model 2: Quadratic	−2.80	0.12	<.001	[−3.03, −2.57]	146,686.2	146,772.9

**Table A2:** Study 2: Sensitivity analysis.

Models	*B*	*SE*	*p*	95% CI	AIC	BIC
Temperature: default						
Study 2: Movie						
Model 1: Linear	0.60	0.02	<.001	[0.57, 0.64]	19,772.4	19,840.9
Model 2: Quadratic	−0.29	0.02	<.001	[−0.33, −0.25]	19,543.7	19,619.1
Temperature: low						
Study 2: Movie						
Model 1: Linear	0.58	0.02	<.001	[0.54, 0.62]	19,858.6	19,927.1
Model 2: Quadratic	−0.29	0.02	<.001	[−0.33, −0.25]	19,628.5	19,703.8
Temperature: high						
Study 2: Movie						
Model 1: Linear	0.44	0.02	<.001	[0.40, 0.48]	21,343.8	21,412.3
Model 2: Quadratic	−0.59	0.02	<.001	[−0.63, −0.55]	20,550.8	20,626.1

## Appendix B. Model-Level Analysis

To further examine whether our findings were not driven by repeated observations from the same model, we conducted supplementary model-level analyses. In particular, we averaged the divergent creativity scores across responses within each language model, such that each model contributed a single observation to the analysis. We then conducted the analyses using these model-level scores.

As shown in Table A3 and Table A4, the quadratic model showed lower AIC and BIC values than the linear model, indicating that the diminishing returns pattern in divergent creativity remained robust in model-level analyses across both studies.

**Table A3:** Study 1: Model-level analysis.

Models	*B*	*SE*	*p*	95% CI	AIC	BIC
Temperature: default						
Study 1: DAT						
Model 1: Linear	2.60	1.14	.029	[0.33, 5.01]	224.0	230.3
Model 2: Quadratic	−3.16	1.08	.006	[−5.27, −1.08]	216.3	224.1
Temperature: low						
Study 1: DAT						
Model 1: Linear	3.62	1.30	.009	[1.05, 6.47]	232.4	238.7
Model 2: Quadratic	−4.05	1.15	.001	[−6.45, −1.83]	221.7	229.4
Temperature: high						
Study 1: DAT						
Model 1: Linear	3.87	1.30	.006	[1.33, 6.48]	233.7	239.9
Model 2: Quadratic	−2.86	1.32	.038	[−5.39, −0.31]	228.8	236.6

**Table A4:** Study 2: Model-level analysis.

Models	*B*	*SE*	*p*	95% CI	AIC	BIC
Temperature: default						
Study 2: Movie						
Model 1: Linear	0.62	0.11	<.001	[0.40, 0.86]	74.2	80.4
Model 2: Quadratic	−0.34	0.10	.002	[−0.54, −0.15]	68.6	76.4
Temperature: low						
Study 2: Movie						
Model 1: Linear	0.61	0.11	<.001	[0.39, 0.83]	71.5	77.8
Model 2: Quadratic	−0.32	0.09	.002	[−0.51, −0.13]	66.5	74.3
Temperature: high						
Study 2: Movie						
Model 1: Linear	0.52	0.18	.008	[0.16, 0.90]	103.4	109.6
Model 2: Quadratic	−0.63	0.16	<.001	[−0.98, −0.32]	94.2	102.0
